# Cultivar-specific coordination of root plasticity and root antioxidant defense underlies drought adaptation in alfalfa

**DOI:** 10.3389/fpls.2026.1941938

**Published:** 2026-10-08

**Authors:** Shuo Li, Guofeng Yang

**Affiliations:** 1 Shandong Key Laboratory for Germplasm Innovation of Saline-Alkaline Tolerant Grasses and Trees, Qingdao Agricultural University, Qingdao, Shandong, China; 2 College of Grassland Science, Qingdao Agricultural University, Qingdao, Shandong, China

**Keywords:** drought resilience, *Medicago sativa* L., root morphological plasticity, root reactive oxygen species, water stress

## Abstract

**Background:**

Alfalfa (*Medicago sativa* L.) is a high-value perennial forage crop whose productivity is severely limited by drought. Although root plasticity and antioxidant defense each contribute to drought tolerance, whether these two dimensions are coordinated or trade off across cultivars remains unclear.

**Method:**

A glasshouse experiment used three alfalfa cultivars (Zhaodong, ZD; Aohan, AH; Golden-empress, GE) and four soil water levels (well water - 75%–80% of water-holding capacity, WHC, mild drought - 60%–65% WHC, moderate - 45%–50% WHC, severe drought - 30%–35% WHC) in a randomized complete block (RCB) design with eight replications per treatment combination. The eight replications were divided into two groups (four for root morphology and four for physiology/biochemistry) to avoid destructive-sampling interference between measurement categories.

**Results:**

ZD and AH reached peak shoot biomass under mild drought, whereas GE exhibited optimal growth under well-watered conditions. In all three cultivars, root biomass, total root length, root surface area and root volume were highest under severe drought, indicating drought-induced root plasticity that potentially enhances soil exploration. MDA, O_2_^-^, H_2_O_2_ increased with drought severity, whereas catalase, ascorbate peroxidase, glutathione peroxidase, glutathione reductase and ascorbic acid showed cultivar-specific modulation. H_2_O_2_ was negatively correlated with ascorbic acid (r = −0.90) and glutathione reductase (r = −0.75).

**Conclusion:**

These findings highlight cultivar-specific strategies for balancing morphological plasticity and biochemical defense. ZD showed the most balanced coordination of root expansion and antioxidant responses, with lower oxidative damage and stable biomass, making it a candidate for breeding programs targeting both drought resilience and yield stability under variable or moderate water availability, whereas GE is better suited to irrigated systems.

## Introduction

Alfalfa (*Medicago sativa* L.) is a perennial forage legume widely recognized as the oldest cultivated forage crop and a dominant and highly desirable component of modern agricultural systems (Benlloch-González et al., 2017; Long et al., 2022). It plays a pivotal role in the livestock industry of semi-arid and arid regions, where its productivity is severely constrained by limited and erratic precipitation. In Northwest of China, with annual rainfall of 200–400 mm and high evaporative demand, which accounts for a substantial proportion of domestic alfalfa production that are representative of water-limited forage production systems worldwide (Hu et al., 2011; Wang et al., 2016). As global climate change intensifies the frequency and severity of drought events, understanding plant adaptive strategies to water deficit has become a critical priority for sustainable agriculture. Drought stress is the primary environmental factor limiting growth and productivity in semiarid regions, triggering a complex suite of morphological, physiological, and biochemical adaptations. As the primary organ for water acquisition, the root system is the first to perceive and respond to soil moisture deficits (Zhao et al., 2023). Root development is governed by both genetic traits and soil environment (Gan et al., 2021). Under sufficient moisture, the root system is mainly distributed in the surface layer of the soil, with less distribution in the deep layer of the soil (Arshad et al., 2018). The distribution of root system can maximize resource utilization; the distribution of roots expansion potentially enhanced soil exploration as inferred from total root length and surface area that are commonly regarded as proxies for soil exploration and water-foraging potential under drought, although direct water-uptake and transport capacity requires root hydraulic measurements (Comas et al., 2013; Lynch, 2013; Shen et al., 2023). Therefore, it is of great significance for improving the drought resistance performance of alfalfa and selecting drought resistant cultivars to investigate the response mechanism of alfalfa root system to drought stress (Clement et al., 2022).

Alfalfa root system has strong plasticity and can adjust its morphological structure and spatial distribution in response to varying soil moisture conditions, thereby enhancing its adaptation to water deficient environments (Pan et al., 2023). Under drought stress, the root system architecture of alfalfa, such as root length, root volume, and root surface area, will change (Ma et al., 2021). These traits are significantly related to drought resistance. In addition to changes in the morphology and structure of roots, plant physiological metabolism has also occurred in plant root system (Li et al., 2017). Under normal environment, the production and scavenging of root reactive oxygen species (ROS) are in a dynamic balance for plants, Concurrently, drought stress induces metabolic shifts, specifically the overproduction of ROS, disrupting the balance (Arshad et al., 2017; Wang et al., 2009, 2024). The ROS cause lipid peroxidation and membrane damage, disrupting cellular integrity (Wang et al., 2024). Alfalfa can regulate antioxidant enzymes such as superoxide dismutase (SOD), catalase (CAT), ascorbate peroxidase (APX), glutathione peroxidase (GPX), and glutathione reductase (GR), as well as non-enzymatic antioxidants including ascorbic acid (AsA), in response to drought stress (Quan et al., 2016; Prince et al., 2022).

Although numerous studies have examined the overall response of alfalfa to water deficit, a critical gap remains in understanding how drought-induced plasticity in root system architecture is associated with antioxidant defense capacity. Previous research often treats these mechanisms as isolated traits, masking how distinct cultivars allocate resources or prioritize specific survival strategies under water-limited conditions. Consequently, it is unclear whether superior drought tolerance stems from morphological priority, physiological priority or their concurrent integration. Moreover, the trade-offs between these two dimensions of adaptation have not been systematically evaluated under controlled drought gradients relevant to Northwest China. Specifically, whereas numerous studies have independently confirmed morphological drought adaptation and physiological (antioxidant) drought tolerance in alfalfa, few have quantified within a single controlled framework whether these two dimensions are positively coupled or trade off, how cultivars differ in their allocation between the CAT/APX and GPX/GR scavenging branches, or whether climatic origin predicts the mode of response.

Therefore, the objectives of this study were: (1) to elucidate the response of root system morphological traits among three alfalfa cultivars with contrasting geographic origins under a gradient of water stress; (2) to measure the shifts in oxidative stress levels and antioxidant defense system; and (3) to compare cultivar-specific strategies in balancing morphological and physiological adaptations. We hypothesize that drought-induced whole-root expansion is positively coupled with antioxidant scavenging capacity, such that cultivars with greater root proliferation also mount a stronger antioxidant response; and cultivars differ in their allocation between the CAT/APX and GPX/GR scavenging branches, with the most drought-adaptive cultivar showing a more balanced deployment across both branches rather than reliance on a single pathway.

## Material and methods

### Plant materials and experimental design

Three alfalfa (*Medicago sativa* L.) cultivars with contrasting geographic origins and adaptation backgrounds were selected: cv. Aohan (AH), cv. Zhaodong (ZD), and cv. Golden-empress (GE). AH originates from arid and semi-arid regions (annual rainfall: 300–400 mm; annual evaporation: ~2,000 mm; mean annual temperature: 6.5 °C). ZD is adapted to semi-arid regions (350–800 mm, annual evaporation of 2,534 mm, 6.4 °C). GE is cultivated in arid regions with moderate moisture and higher temperature requirements (400–600 mm; 1,500 mm; 8.7 °C). Based on their climatic zones of origin, cultivars were selected to span a gradient of climatic origin and putative drought-adaptation background, in order to test whether geographic origin predicts the mode of drought response. The experiment was conducted from March to September 2021 in a controlled-environment greenhouse under regulated temperature, relative humidity, and photoperiodic conditions at the Institute of Animal Science, Chinese Academy of Agricultural Sciences, Beijing, China. Plants were grown under a photoperiod cycle of 16 h of light followed by 8 h darkness. During the experimental period, the diurnal temperature fluctuated between a maximum of 28 °C and a minimum of 20 °C, with an average daytime temperature maintained at 25.3 ± 2 °C. Supplemental lighting was provided by high-pressure sodium lamps, ensuring a photosynthetic photon flux density of approximately 400–600 μmol·m^-2^·s^-1^ at the canopy level. Average relative humidity was maintained at 50%–70% throughout the growth cycle. To avoid positional effects, pots were rearranged weekly throughout the duration of the experiment. Prior to sowing, uniform alfalfa seeds were selected and surface-disinfected in 1% (v/v) sodium hypochlorite (NaClO) solution for 15 min. Then rinse them three times with distilled water. A seed was transferred to a cylindrical PVC tube (50 cm in height and 18 cm in diameter) containing 3 kg of cultivation substrate with an internal nylon mesh bag, with one plant established per tube. The cultivation substrate consisted of a 2:1:1 (v/v/v) mixture of loamy field soil, peat moss, and vermiculite. All three cultivars possess a taproot system with abundant lateral roots, not a tillering root system.

### Determination of water holding capacity

Water holding capacity for each pot was determined by weighing the dry pot, watering until it dripped, letting the pot stand 48 h, and weighing it again. The difference between the dry and next-day weight of each pot was recorded as pot capacity.

At the start of the experiments, pots with dry potting mix were weighed and then watered until they were fully saturated. They were then drained for 48 h, and individual pots were weighed again to determine water holding capacity (moisture content at this point), which were used as the basis to calculate drought stress levels of the treatments.

### Drought stress treatments and soil moisture control

When seedlings reached the age of 2 months (early vegetative stage, 6–8 trifoliate leaves expanded), a 4-week drought stress treatment was initiated. The experiment comprised a 3 (cultivar) × 4 (soil moisture) factorial design arranged in a randomized complete block (RCB) with 8 replicates (per treatment combination (n = 4 for root morphological measurements; n = 4 for physiological and biochemical assays). Soil moisture treatments were defined as percentages of field water-holding capacity (WHC): (1) 75%–80% WHC (well-watered control, CK); (2) 60%–65% WHC (mild drought); (3) 45%–50% WHC (moderate drought); and (4) 30%–35% WHC (severe drought). During the period of experiment, the irrigation was withheld from the drought-treated tubes until the soil moisture in each tube declined to its designated percentage of water-holding capacity (WHC). The well-watered control was consistently maintained at 75%–80% WHC throughout the experimental period. Once soil moisture reached its target range, soil moisture was maintained gravimetrically for the 4-week treatment period. Each PVC tube was weighed, and water was added to restore the target WHC for each treatment. Target weights for each moisture treatment were calculated based on the dry weight of the substrate in each tube, ensuring that each pot was maintained within ± 2% of its assigned WHC range. To avoid positional effects within blocks, pots were rearranged weekly within their assigned block throughout the duration of the experiment. At the end of experiment, each trait was measured on n = 4 independent plants per treatment combination (four plants for root morphological measurements and four plants for physiological/biochemical assays); the two measurement categories were kept independent to avoid destructive-sampling interference. Sample for physiological analyses was quickly placed in liquid nitrogen immediately after collection and stored at -80 °C until analysis.

### Measurements

#### Root morphology and biomass measurements

The nylon mesh bags were carefully removed from each PVC tube to minimize disturbance to the spatial distribution of the root system. Roots were gently washed with deionized water and evenly spread in a transparent acrylic tray containing 1 cm of distilled water (**Supplementary Figure 1**). Root systems were scanned at 600 dpi resolution using a MICROTEK Scan Maker i800 plus. Images were analyzed with WinRHIZO 2017a software (Regent Instruments, Inc., Quebec, QC, Canada), including threshold conversion, framing, editing of breakpoints, and loop elimination to quantify root length (cm), root surface area (cm²), average root diameter (mm), and root volume (cm³). After scanning, root and shoot samples were oven-dried at 65 °C until constant weight was attained. Root and shoot biomass were calculated as g DM per pot based on the dry matter weight per pot. For biochemical assays, the entire root system (including the taproot and attached lateral roots, without separation into fine and coarse root fractions) was pooled per plant.

### Detection of H_2_O_2_ and O_2_^-^

Soluble protein in the enzyme extract was quantified by the Bradford method using bovine serum albumin (BSA) as the standard, with absorbance read at 595 nm. A BSA standard curve (0–20 µg mL^-1^) was constructed for each plate, and the measured protein concentration was used to normalize every enzyme activity to U/mg protein. Hydrogen peroxide (H_2_O_2_) content was determined spectrophotometrically at 415 nm following the method of Willekens et al. (1997). Roots (0.1 g) were homogenized in 4 mL 0.1% (w/v) TCA in an ice bath and the extract was centrifuged at 12,000 × g for 10 min at 4 °C. The supernatant was reacted with 10 mM potassium phosphate buffer (pH 7.0), and the absorbance of the triiodide product was read at 415 nm after a 20 min incubation in the dark. H_2_O_2_ concentration was calculated from a standard curve prepared with known H_2_O_2_ concentrations and expressed as mmol g^-1^ protein. The rate of O_2_^-^ generation was measured according to Elstner and Adelheid (1976). Root (0.1 g) was homogenized in 4 mL of 65 mM phosphate buffer (pH 7.8) at 25 °C for 1 h, and centrifuged at 5,000 × g for 15 min at 4 °C. The absorbance of the supernatant was measured at 530 nm after the addition of an equal volume of ether and centrifugation at 3,000 × g for 3 min. O_2_^-^ generation was calculated against a NaNO_2_ standard curve and expressed as U/mg protein.

### Determination of malondialdehyde

MDA concentration, as a marker of lipid peroxidation, was determined using the modified method of Heath and Packer (1968). Root samples (0.5 g) were ground in liquid nitrogen and homogenized with 5 mL of 20% (w/v) trichloroacetic acid (TCA). Absorbance was measured at 450, 532, and 600 nm using a spectrophotometer (NanoDrop 2000), and MDA concentration was calculated after correcting for non-specific turbidity.

### Antioxidant enzyme extraction and activity assays

Root samples (0.5 g) were ground in liquid nitrogen using a pre-chilled mortar and pestle. Homogenization was performed with 5 mL of ice-cold extraction buffer consisting of 100 mM potassium phosphate-buffered saline (PBS) and 0.1 mM Na_2_EDTA (pH 7.0–7.4). The homogenate was filtered through four layers of muslin cloth and centrifuged at 16,000 × g for 20 min at 4 °C. The supernatant was used as the enzyme extract for all activity assays. All extraction procedures were conducted at 4 °C; enzymatic activity measurements were performed at 25 °C.

Catalase (CAT) activity was assayed following Durner and Klessig (1996). Enzyme extract (100 μL) was mixed with 3 mL of 0.15 M PBS and 5 μL of 0.1 M H_2_O_2_. The decrease in absorbance at 240 nm was recorded. One unit of CAT was defined as the amount of enzyme that decomposed 1 μmol of H_2_O_2_ per minute under the assay conditions.

Ascorbate peroxidase (APX) activity was measured according to Nakano and Asada (1980). The reaction mixture (2 mL final volume) contained 50 mM PBS (pH 7.0), 0.5 mM ascorbic acid, 0.1 mM EDTA, 0.1 mM H_2_O_2_, and 30 μL of enzyme extract. Activity was calculated from the decrease in absorbance at 290 nm over 1 min at room temperature. One unit of APX was defined as the amount of enzyme that oxidized 1 μmol of ascorbate per minute under the assay conditions.

Ascorbic acid (AsA) concentration was determined spectrophotometrically according to the method of Kampfenkel et al. (1995). Root tissue was extracted with 6% TCA and centrifuged at 15,000 × g for 15 min at 4°C, and the supernatant was used for AsA assays. The supernatant (0.2 mL) was neutralized with 0.5 mL of 0.2 M K-phosphate buffer (pH 7.4) and 0.1 mL dithiothreitol (DTT). The reaction mixture was incubated at 30°C for 90 min, and the concentration was determined spectrophotometrically at 525 nm.

Glutathione peroxidase (GPX) activity was assayed according to Elia et al. (2003). The reaction mixture contained 100 mM PBS (pH 7.0), 1 mM EDTA, 1 mM NaN_3_, 0.12 mM NADPH, 2 mM GSH, 1 U glutathione reductase, 0.6 mM H_2_O_2_ (as substrate), and 30 μL of enzyme extract. The reaction was initiated with H_2_O_2_, and the decrease in absorbance was measured at 340 nm. One unit of GPX was defined as the amount of enzyme that oxidized 1 μmol of NADPH per minute under the assay conditions.

Glutathione reductase (GR) activity was assayed following Foyer and Halliwell (1976) and Cakmak and Marschner (1992). The reaction mixture contained 0.1 M PBS (pH 7.0), 1 mM EDTA, 1 mM GSSG, 0.2 mM NADPH, and 50 μL of enzyme extract. The reaction was initiated by the addition of GSSG, and the decrease in absorbance at 340 nm was recorded. One unit of GR was defined as the amount of enzyme that oxidized 1 μmol of NADPH per minute under the assay conditions.

### Statistical analysis

Data were analyzed using general analysis of variance (ANOVA) in GENSTAT (GENSTAT Committee 2019). A randomized complete block (RCB) design model (cultivar and water stress as fixed factors and block as a random factor) was used to analyze shoot and root biomass, root morphological traits, reactive oxygen species, and antioxidant enzyme activities and non-enzymatic antioxidants for each cultivar among water stress treatments. A randomized complete block (RCB) design model with 3 × 4 = 12 factorial treatment was used to analyse their interaction on root traits and root physiology. Principal component analysis (PCA) was performed on the correlation matrix so that traits on different scales contributed equally. Pairwise relationships were assessed with Pearson product-moment correlations. Treatment means were compared using Fisher’s protected least significant difference (LSD) test. Significant differences were denoted as *p* < 0.05 or *p* < 0.01. All figures and tables present mean values ± standard error (SE).

## Results

### Shoot and root biomass

There were significant (p < 0.05) differences on shoot biomass for AH and GE among various water stress (**Table 1**). Shoot biomass of AH was the highest under mild drought (60%–65% WHC), reaching 3.90 g DM/pot. GE showed the highest shoot biomass under well-watered control, with the sequences of mild drought > moderate drought > severe drought. Overall, there was an upward trend on shoot biomass for GE with the increasing of soil water content (**Table 1**). There was no significant difference on shoot biomass for ZD among various water treatments, ranging from 3.58 to 3.80 g DM/pot (**Table 1**).

**Table 1:** Shoot biomass, root biomass and root/shoot ratio of three alfalfa cultivars under four soil moisture treatments.

Cultivars	Water stress			
	Severe drought 30%-35% WHC	Moderate drought 45%-50% WHC	Mild drought 60%-65% WHC	Well-watered 75%-80% WHC
Shoot biomass (g DM/pot)				
ZD	3.58 ± 0.265a	3.61 ± 0.072a	3.80 ± 0.1176a	3.73 ± 0.291a
AH	2.96 ± 0.236c	3.05 ± 0.087c	3.90 ± 0.170a	3.49 ± 0.053b
GE	3.44 ± 0.090b	3.43 ± 0.072b	4.14 ± 0.164a	4.26 ± 0.186a
Root biomass (g DM/pot)				
ZD	0.41 ± 0.005a	0.38 ± 0.017a	0.27 ± 0.028b	0.30 ± 0.010b
AH	0.48 ± 0.044a	0.38 ± 0.022b	0.24 ± 0.012d	0.30 ± 0.018c
GE	0.52 ± 0.020a	0.38 ± 0.077b	0.34 ± 0.038bc	0.26 ± 0.055c
Root/shoot ratio				
ZD	0.11 ± 0.007a	0.11 ± 0.002a	0.07 ± 0.005c	0.08 ± 0.005b
AH	0.16 ± 0.020a	0.13 ± 0.009b	0.06 ± 0.006d	0.08 ± 0.004c
GE	0.15 ± 0.005a	0.11 ± 0.024b	0.08 ± 0.011bc	0.06 ± 0.014c

Values are means ± SE. Different letters within a row indicate significant differences among water treatments at 0.05 level.

There was a significant (p < 0.05) difference on root biomass for ZD among various water stress (**Table 1**). When soil water content was under severe drought, root biomass was the highest (0.41 and 0.48 g DM/pot) for ZD (0.41 g DM/pot) and AH (0.48 g DM/pot). GE reached its maximum root biomass under severe drought (0.52 g DM/pot), with a consistent downward trend as soil water content increased. Overall, there was a downward trend on root biomass of GE with increasing of soil water content (**Table 1**).

The ratio of root to shoot was the lowest for ZD and AH under mild drought conditions, at 0.07 and 0.06, respectively (**Table 1**). When soil water was under well water conditions, root to shoot ratio was the lowest (0.06) for GE (**Table 1**).

### Total root length

Total root length of ZD was the highest (660.1 cm) when soil water content was under severe drought, being more 57.9 cm, 116.1 cm and 33.6 cm greater than moderate drought, mild drought and well-water control, respectively (**Table 2**). There was a significant (p < 0.05) difference on total root length for ZD, AH and GE, respectively (**Table 2**). Similarly, AH showed the similar trend to compare ZD. Total root length of GE was the highest (821.1 cm) when soil water content was under severe drought, with the sequence of moderate drought > mild drought > well-watered control. Overall, there was a downward trend for total root length of GE with the increasing of soil water content (**Table 2**).

**Table 2:** Total root length (cm) of three alfalfa cultivars under four soil moisture treatments.

Cultivars	Water stress			
	Severe drought 30%-35% WHC	Moderate drought 45%-50% WHC	Mild drought 60%-65% WHC	Well-watered 75%-80% WHC
ZD	660.1 ± 16.71a	602.2 ± 33.34ab	544.0 ± 62.34b	626.5 ± 78.11ab
AH	759.4 ± 14.69a	692.4 ± 54.55b	494.3 ± 21.85d	612.1 ± 44.30c
GE	821.1 ± 109.09a	644.4 ± 27.40b	549.0 ± 9.86b	420.4 ± 19.24c

Values are means ± SE. Different letters within a row indicate significant differences among water treatments at 0.05 level.

### Root surface area

When soil water content was under severe drought, root surface area of ZD was the highest (69.3 cm^2^), 25.5% than mild drought (**Table 3**). To compare ZD, AH showed the similar trend on root surface area. There was a downward trend on root surface area for GE with the increasing of soil water content (**Table 3**).

**Table 3:** Root surface area (cm^2^) of three alfalfa cultivars under four soil moisture treatments.

Cultivars	Water stress			
	Severe drought 30%-35% WHC	Moderate drought 45%-50% WHC	Mild drought 60%-65% WHC	Well-watered 75%-80% WHC
ZD	69.3 ± 0.46a	64.7 ± 4.60ab	55.2 ± 16.04b	60.5 ± 7.95ab
AH	77.3 ± 0.69a	74.7 ± 2.38a	55.5 ± 2.45c	57.9 ± 0.60b
GE	80.2 ± 6.47a	63.7 ± 3.90b	60.8 ± 3.38b	48.4 ± 6.40c

Values are means ± SE. Different letters within a row indicate significant differences among water treatments at 0.05 level.

### Average root diameter

When soil water content was under severe drought, average root diameter of ZD and AH were the highest, at 0.42 mm and 0.37 mm, respectively (**Table 4**). When soil water content was under mild drought, the average root diameter was lower of ZD and AH, at 0.34 mm and 0.33 mm, respectively. There was a downward trend on average root diameter for GE with the increasing of soil water content (**Table 4**).

**Table 4:** Average root diameter (mm) of three alfalfa cultivars under four soil moisture treatments.

Cultivar	Water stress			
	Severe drought 30%-35% WHC	Moderate drought 45%-50% WHC	Mild drought 60%-65% WHC	Well-watered 75%-80% WHC
ZD	0.42 ± 0.004a	0.36 ± 0.026b	0.34 ± 0.019b	0.35 ± 0.016b
AH	0.37 ± 0.009a	0.34 ± 0.012ab	0.33 ± 0.022b	0.35 ± 0.011ab
GE	0.38 ± 0.009a	0.38 ± 0.018a	0.39 ± 0.020a	0.35 ± 0.017b

Values are means ± SE. Different letters within a row indicate significant differences among water treatments at 0.05 level.

### Root volume

When soil water content was under mild drought, root volume of ZD and AH was less than severe drought, moderate drought and well water (**Table 5**). Root volume for GE was the highest (0.98 cm^3^) when soil water content was under severe drought, was 19.5%, 88.5% and 127.9% greater than moderate drought, mild drought and well water. There was a downward trend on root volume for GE with the increasing of soil water content (**Table 5**).

**Table 5:** Root volume (cm^3^) of three alfalfa cultivars under four soil moisture treatments.

Cultivars	Water stress			
	Severe drought 30%-35% WHC	Moderate drought 45%-50% WHC	Mild drought 60%-65% WHC	Well-watered 75%-80% WHC
ZD	0.65 ± 0.025a	0.52 ± 0.008ab	0.42 ± 0.038b	0.54 ± 0.124a
AH	0.65 ± 0.127a	0.65 ± 0.062a	0.44 ± 0.055b	0.51 ± 0.024b
GE	0.98 ± 0.042a	0.82 ± 0.064b	0.52 ± 0.050c	0.43 ± 0.035c

Values are means ± SE. Different letters within a row indicate significant differences among water treatments at 0.05 level.

### MDA concentration

When soil water content was under severe drought, ZD was at the highest (13.8 nmol/mgprot) on MDA concentration, followed by moderate drought (13.3), mild drought (10.6) and well water (7.6 nmol/mgprot). Overall, the concentration of MDA of ZD showed a downward trend with the increasing of soil water content. Both AH and GE on the MDA concentration also showed downward trends with increasing of soil water content (**Table 6**).

**Table 6:** MDA concentration (nmol/mgprot) in root system of three alfalfa cultivars under four soil moisture treatments.

Cultivar	Water stress			
	Severe drought 30%-35% WHC	Moderate drought 45%-50% WHC	Mild drought 60%-65% WHC	Well-watered 75%-80% WHC
ZD	13.8 ± 0.44a	13.3 ± 0.21a	10.6 ± 0.88b	7.6 ± 0.53c
AH	15.9 ± 0.62a	14.7 ± 0.17b	12.9 ± 0.31c	10.5 ± 0.42d
GE	14.9 ± 0.35a	14.3 ± 0.71a	13.1 ± 0.32b	12.4 ± 0.35b

Values are means ± SE. Different letters within a row indicate significant differences among water treatments at 0.05 level.

### Reactive oxygen species (O_2_^-^ and H_2_O_2_)

There were significant (p < 0.05) differences on O_2_^-^ concentration for ZD and GE among various water stress (**Table 7**). There were downward trends for all cultivars with the increasing of soil water content. The O_2_^-^ concentration was the highest for ZD, AH and GE when soil water content was under severe drought, at 3.0, 3.5 and 3.6 U/mgprot, respectively (**Table 7**).

**Table 7:** Superoxide anion O_2_^-^ and hydrogen peroxide H_2_O_2_ concentration in root system of three alfalfa cultivars under four soil moisture treatments.

Cultivars	Water stress			
	Severe drought 30%-35% WHC	Moderate drought 45%-50% WHC	Mild drought 60%-65% WHC	Well-watered 75%-80% WHC
O_2_^-^ (U/mgprot)				
ZD	3.0 ± 0.09a	2.5 ± 0.09b	1.9 ± 0.06c	1.8 ± 0.04d
AH	3.5 ± 0.12a	2.5 ± 0.03b	2.4 ± 0.09bc	2.3 ± 0.03c
GE	3.6 ± 0.11a	3.1 ± 0.08b	2.7 ± 0.09c	2.5 ± 0.01d
H_2_O_2_ (mmol/gprot)				
ZD	20.2 ± 0.89a	13.8 ± 0.54b	11.1 ± 0.71c	8.2 ± 0.94d
AH	36.9 ± 0.46a	35.1 ± 1.11a	23.3 ± 4.04b	14.7 ± 1.14c
GE	39.3 ± 3.03a	29.7 ± 0.95b	20.9 ± 1.44c	13.0 ± 1.36d

Values are means ± SE. Different letters within a row indicate significant differences among water treatments at 0.05 level.

There were significant (p < 0.05) differences on H_2_O_2_ concentration for ZD and GE among various soil water content. The H_2_O_2_ concentration showed downward trends with the increasing of soil water content for all cultivars. GE was the highest on H_2_O_2_ concentration (39.3 mmol/gprot) when soil water content was under severe drought, followed by AH (36.9 mmol/gprot) > ZD (20.2 mmol/gprot). The H_2_O_2_ concentration showed a similar trend for GE, AH and ZD under well water.

### Antioxidant enzyme and ascorbic acid

There were downward trends with the increasing of soil water content for all cultivars (**Figure 1a**). CAT declined from 6.8 U/mgprot to 3.3 U/mgprot, from 5.2 U/mgprot to 2.8 U/mgprot and from 8.5 U/mgprot to 4.6 U/mgprot for ZD, AH and GE, respectively. There were significant (p < 0.05) differences on the activity of APX for ZD and GE among various water stress (**Figure 1b**). The activity of APX decreased from 0.42 U/mgprot to 0.23 U/mgprot, from 0.34 U/mgprot to 0.24 U/mgprot, from 0.49 U/mgprot to 0.38 U/mgprot for ZD, AH and GE (**Figure 1b**).

**Figure 1:**
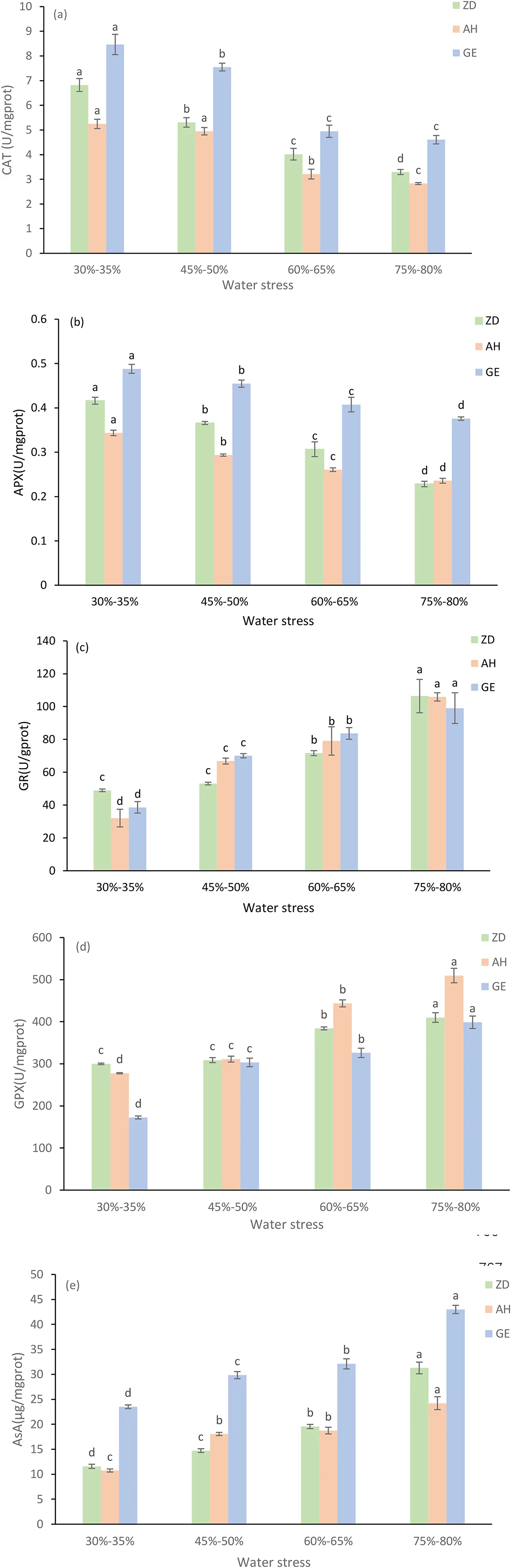
The concentration on **(a)** CAT, **(b)** GR **(c)** GPX, **(d)** APX and **(e)** AsA in root system of three alfalfa cultivars under four soil moisture treatments. Different letters compare water treatments within a cultivar Values are means ± SE. Different letters indicate significant differences among water treatments at 0.05 level.

There were significant (p < 0.05) differences on GR activity for AH and GE among various water stress. The activity of GR ranged from 49.0 U/gprot to 106.4 U/gprot for ZD, ranged from 32.1 U/gprot to 105.9 U/gprot for AH, and ranged from 38.6 U/gprot to 99.1 U/gprot for GE (**Figure 1c**). There were significant differences (p < 0.05) on for AH and GE among various water stress. GPX activity of ZD, AH and GE was the highest, at 410.0 U/mgprot, 509.7 U/mgprot and 398.7 U/mgprot when soil water content was at the 75%-80% of WHC, respectively, followed by 60%-65%, 45%-50% and 30%-35% of WHC. (**Figure 1d**).

There were significant (p < 0.05) differences on AsA concentration for ZD, AH and GE among various water stress (**Figure 1e**). There were upward trends on AsA concentration for ZD, AH and GE with the increasing of soil water content. The content of AsA ranged from 11.6 to 31.3 µg/mgprot for ZD, ranged from 10.8 to 24.2 µg/mgprot for AH. And ranged from 23.5 µg/mgprot to 43.0 µg/mgprot for GE (**Figure 1e**).

### Principal component analysis and inter-trait correlations

The PCA ordination diagram showed that whole-root system characteristics and root physiological functions differed among various water stress treatments (**Figure 2**). The 95% confidence ellipses showed that cultivar separation occurs mainly along PC2. Drought stress spread along PC1 reflects the drought-severity gradient, the data points for each water stress treatment for each cultivar were clustered (**Figure 2**). The PC1 and PC2 axes explained 74.9% of the total variation under the water deficit treatments (**Figure 2**). PC1 is driven by root biomass (9.37%), GPX (9.02%), root/shoot ratio (9.03%), GR (9.01%), O_2_^−^ (8.68%) and CAT (7.74%); PC2 is dominated by AsA (22.11%), APX (19.00%) and H_2_O_2_ (17.17%), followed by shoot biomass (12.91%) (**Supplementary Table 1**).

**Figure 2:**
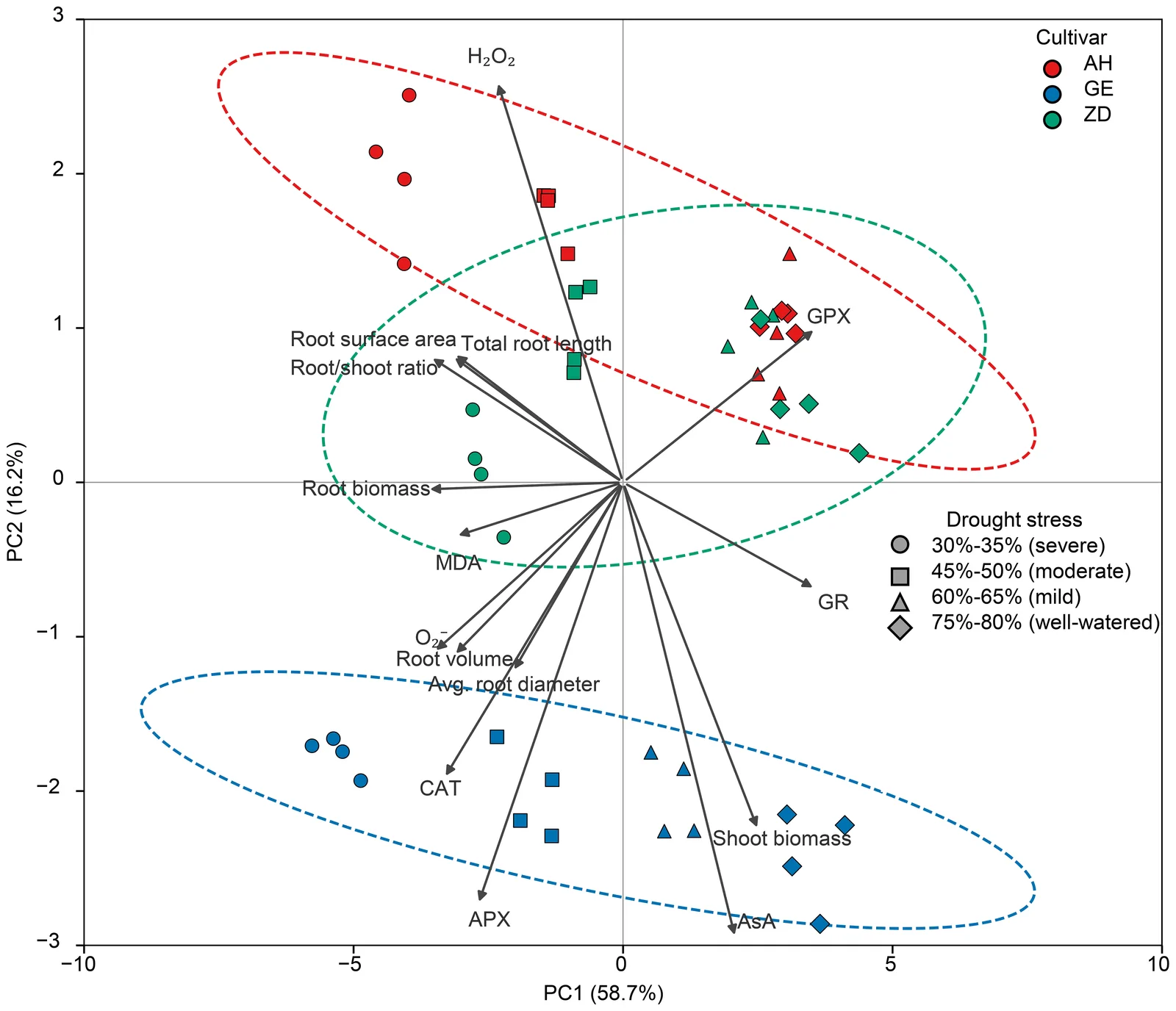
Principal component analysis (PCA) biplot of 48 root system samples. Points are colored by cultivar (AH red, GE blue, ZD green) and shaped by drought severity; ellipses are 95% confidence regions per cultivar. The separation of ellipses along PC2 reflects the cultivar-driven biochemical variation, whereas the spread along PC1 reflects the drought-severity gradient common to all cultivars. Arrows are variable loadings on the PC1–PC2 plane.

Root biomass was positively correlated with total root length (r = 0.76, p < 0.001), root volume (r = 0.69, p < 0.001) and root surface area (r = 0.69, p < 0.001). Total root length was positively correlated with root volume (r = 0.78, p < 0.001) and root surface area (r = 0.74, p < 0.001). Shoot biomass was negatively correlated with total root length (r = −0.67, p < 0.001) and root biomass (r = −0.57, p < 0.001), and positively correlated with AsA (r = 0.60, p < 0.001). GPX was positively correlated with GR (r = 0.81, p < 0.001), but negatively with CAT (r = −0.87, p < 0.001), APX (r = −0.79, p < 0.001) and O_2_^−^ (r = −0.76, p < 0.001). CAT was positively correlated with APX (r = 0.92, p < 0.001). H_2_O_2_ was positively correlated with O_2_^-^ (r = 0.80, p < 0.001) and with MDA (r = 0.80, p < 0.001, **Figure 3**).

**Figure 3:**
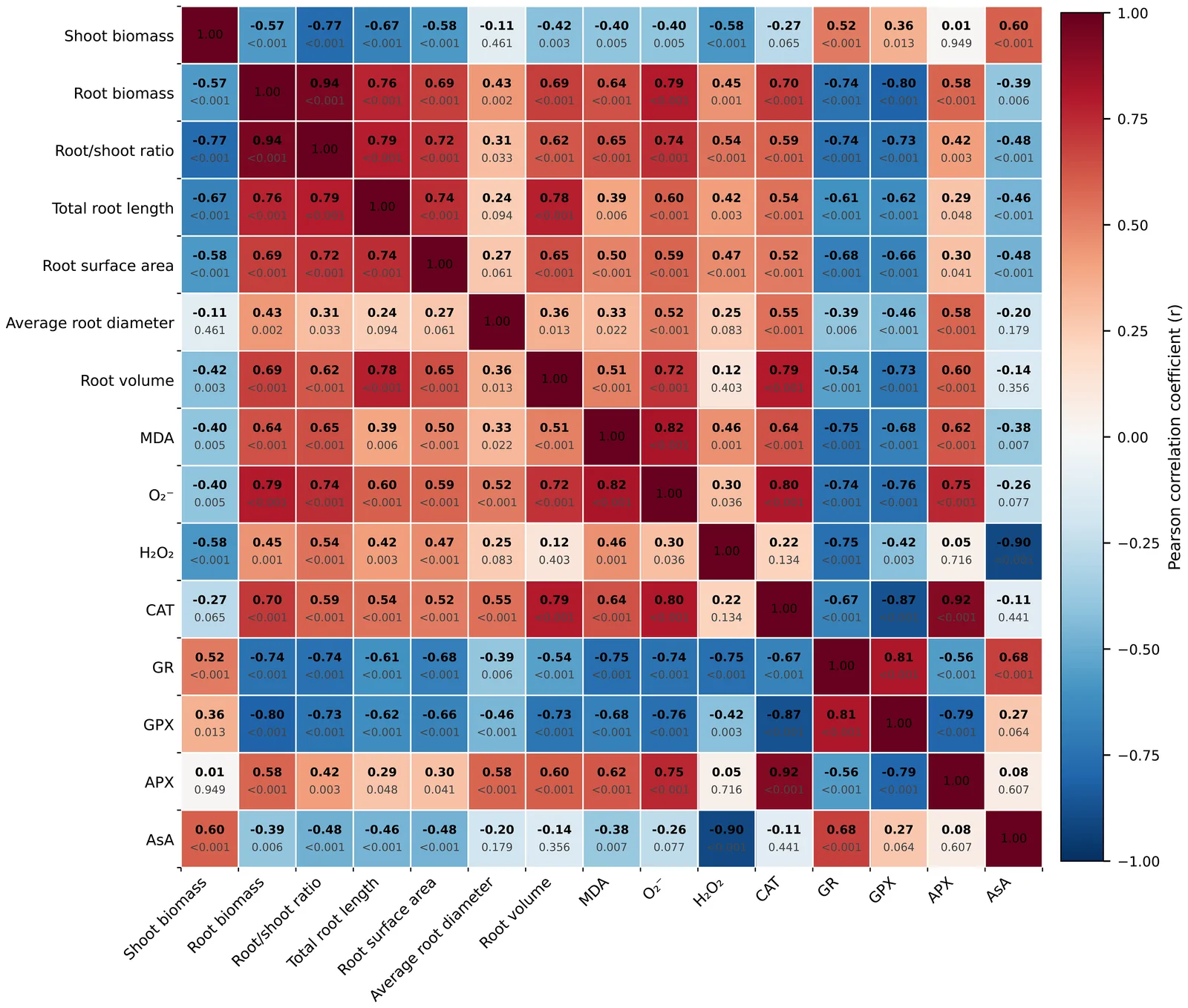
Pearson correlation analysis of the 15 traits. The heatmap displays the Pearson correlation coefficient (upper value) and the corresponding p-value (lower value); p-values below 0.001 are indicated as <0.001, and the diagonal is left blank for p. Red indicates positive and blue negative correlation, as indicated by the color key on the right.

### Interaction effect between cultivars and water stress

There were significant (p < 0.05, 0.01) interactions between alfalfa cultivars and water stress on root biomass, shoot biomass, root/shoot ratio, total root length, root surface area, average root diameter and root volume (**Table 8**). There were significant (p < 0.01, 0.01) interactions on root trait and root physiology except O_2_^-^ and APX between alfalfa cultivars and water stress (**Table 8**). Water stress treatment had significant (p < 0.01) effects on root trait and root physiology except APX. Alfalfa cultivar had significant (p < 0.05, 0.01) effect on MDA, GPX, CAT, O_2_^-^, H_2_O_2_, APX, root biomass and root volume (**Table 8**).

**Table 8:** Results of two-way ANOVA of plant species and drought treatment effect on 15 measured traits.

Category	Variable	Cultivars			Water stress			Interaction		
		F	df	P	F	df	P	F	df	P
Physiology	MDA	110.0	2	< 0.001	78.74	3	< 0.001	15.55	6	< 0.001
	GPX	156.3	2	< 0.001	290.1	3	< 0.001	41.83	6	< 0.001
	CAT	242.8	2	< 0.001	293.7	3	< 0.001	9.576	6	< 0.001
	O_2_^-^	483.6	2	< 0.001	278.1	3	< 0.001	0.802	6	0.506
	GR	0.962	2	0.337	111.1	3	< 0.001	9.143	6	< 0.001
	H_2_O_2_	390.3	2	< 0.001	101.5	3	< 0.001	21.32	6	< 0.001
	AsA	1561.1	2	< 0.001	649.6	3	< 0.001	6.001	6	0.004
	APX	27.30	2	< 0.001	1.792	3	0.178	1.751	6	0.186
Root system	Root biomass	4.493	2	0.046	19.69	3	< 0.001	3.370	6	0.037
	Shoot biomass	2.860	2	0.105	10.78	3	< 0.001	4.584	6	*0.012
	Root/shoot ratio	3.051	2	0.095	28.02	3	< 0.001	5.896	6	0.004
	Total root length	1.121	2	0.301	15.60	3	< 0.001	11.48	6	< 0.001
	Root surface area	0.501	2	0.486	8.486	3	0.001	3.182	6	0.044
	Average root diameter	0.601	2	0.447	8.385	3	0.001	4.656	6	0.011
	Root volume	557.9	2	0.000	34.68	3	< 0.001	14.95	6	< 0.001

Reported statistics include F-values and P-values for cultivars (df = 2), water stress (df = 3) and their interaction (df = 6). **, * mean F-value significant at p < 0.01 and p < 0.05, respectively; ns means no significance.

## Discussion

### Cultivar-specific biomass allocation reflects contrasting drought adaptation strategies

In recent decades, water has been considered a critical environmental factor, and its deficiency is known as drought stress which leads to restricted plant growth, yield, and quality, mainly due to various morphological, physiological, and biochemical responses (Lamb et al., 1995; Zhu et al., 2005; Li et al., 2022; Huang et al., 2019). A controlled drought gradient was applied to three alfalfa cultivars with contrasting geographic origins. ZD and AH achieved peak shoot productivity under mild drought, while GE showed a linear increase in shoot biomass with increasing soil moisture. These patterns are consistent with the cultivars’ native environments. Root biomass responded inversely to shoot biomass across cultivars, reflecting belowground- aboveground partitioning trade-offs. This shift in biomass partitioning aligns with the well-established paradigm of drought-induced preferential carbon allocation to whole-root system, which potentially enhances soil exploration and water foraging capacity, the magnitude differed among cultivars, with ZD maintained a more stable root-to-shoot allocation across treatments, whereas AH exhibited the largest shift and GE was intermediate under water stress, suggesting that GE relies more heavily on morphological plasticity, whereas ZD balances morphological and biochemical strategies more evenly. These findings are consistent with genotype-specific biomass allocation reported in alfalfa (Ma et al., 2021; Prince et al., 2022). Our results extend these findings by demonstrating that the balance between root morphological and biochemical strategies differs predictably based on the cultivar’s climatic origin.

### Drought-induced root system plasticity potentially enhances soil exploration capacity

The root system is the primary organ that perceives and responds to changes in soil moisture. Root morphology serves as a pivotal indicator for predicting plant responses and adaptations to changing environments (Xu et al., 2023). Under drought conditions, root physiology and architecture are substantially affected, ultimately influencing water and nutrient uptake capacity (Atkinson et al., 2019; Lu et al., 2019; Fromm, 2019). In the present study, the four-week stress treatment was applied to young vegetative-stage plants. All cultivars exhibited optimal root morphological development under severe drought, with significant increases in total root length, root surface area, root volume, consistent with the drought-induced root proliferation paradigm (Li et al., 2017). The 25%–54% increase in root volume under severe drought relative to mild drought conditions across cultivars is comparable in magnitude to values reported by Li et al. (2017) for alfalfa subjected to progressive drought. In the present study, root diameter was also more for ZD, AH and GE when soil water content was 30%-35% of WHC than 45%-50%, 60%-65% and 75%-80% of WHC. These results indicated that drought could promote the increase of average root diameter, which might serve as a protective strategy for plants to alleviate drought stress. The increase in average root diameter under severe drought observed in ZD and AH may represent a protective anatomical strategy. Thicker roots are generally associated with a larger stele and cortex (Jeong et al., 2013; Stefan and Peter, 2002), which may enhance water transport efficiency and provide greater storage capacity for osmotically active solutes.

### Regulation of ROS production and antioxidant defense under water stress

Drought induces excess generation of ROS in plants, causing oxidative damage to membranes and impairing photosynthesis, mineral absorption, and assimilation (Chen et al., 2022; Yang et al., 2021; Mittler et al., 2011; Fichman and Mittler, 2020; Ali et al., 2025). In the present study, H_2_O_2_, O_2_^-^ and malondialdehyde (MDA) were the highest under severe drought and declined with the increasing of soil moisture (Li et al., 2020; Zhang and Shi, 2018). In our study, ZD maintained lower H_2_O_2_, O_2_^-^ and MDA concentration than AH and GE across treatments, suggesting more effective ROS scavenging that limits secondary membrane damage. In order to reduce stress-triggered ROS accumulation, plants have developed a complex array of several enzymatic and non-enzymatic antioxidant protection mechanisms against the damaging effects of ROS under environmental stresses (Souza et al., 2014; Pallavi et al., 2012). In the present study, CAT and APX activity was highest under severe drought and declined with increasing soil moisture, consistent with their role as a high-capacity H_2_O_2_ scavenger that serves as a cellular sink for photorespiratory and stress-induced H_2_O_2_ (Gechev et al., 2002; Gabara et al., 2003). By contrast, GR and GPX were the lowest under severe drought and rose with soil moisture. This inverse relationship reflects complementary regulation of two interlinked H_2_O_2_-scavenging pathways: under drought the ascorbate–peroxidase branch (CAT/APX) is upregulated while the glutathione branch (GPX/GR) is drawn down as its reductant pool is consumed. These traits are consistent with the strong negative correlations between GPX and the peroxidases (GPX–CAT r = −0.87; GPX–APX r = −0.79) and the positive CAT–APX correlation (r = 0.92), indicating that the two H_2_O_2_-scavenging pathways are coordinated. GE mounted the strongest ascorbate-peroxidase response (highest CAT and APX) also accumulated the highest H_2_O_2_ and MDA, indicating that its enzyme induction was insufficient to fully offset oxidative load; AH showed the opposite trend (highest GPX, lowest CAT/APX). ZD maintained intermediate activities across both branches. The negative correlation between H_2_O_2_ and AsA (r = −0.90) was the biochemical signature of this pattern, where oxidative load is high, the ascorbate pool is consumed in detoxification, whereas under relaxed conditions the pool is replenished in its reduced form.

### Integrated coordination of root architecture and antioxidant defense defines cultivar-specific drought adaptation

A key objective of this study was to move beyond treating root morphology and antioxidant defense as independent traits and instead evaluate their concurrent regulation. The enzyme data and correlations indicate that drought adaptation is best understood as the coordinated deployment of a root-proliferation module and one of two inversely related antioxidant branches. ZD exhibited a balanced strategy: moderate root system expansion was coupled with intermediate activities across both the CAT/APX and GPX/GR branches, which was associated with lower MDA and stable shoot biomass. AH, despite the strongest glutathione branch, showed the weakest peroxidases (lowest CAT, APX and AsA) and accumulated higher MDA, indicating that its reliance on a single branch did not fully offset oxidative damage. GE combined the most pronounced root plasticity with the strongest ascorbate-peroxidase response (highest CAT, APX and AsA), but its lowest GPX and highest MDA suggest that a defense skewed toward the CAT/APX–ascorbate axis is less flexible when ascorbate becomes limiting. This integrated framework is consistent with the emerging concept that drought adaptation is an emergent property of concurrent phenotypic integration, rather than the sum of independent trait responses. The findings align with previous work by Li et al. (2020) and Quan et al. (2016) demonstrating that alfalfa root systems characteristics are coupled with root antioxidant capacity under water treatment regimes. It is important to note that the two categories of traits operate on different timescales. Root morphological traits integrate the cumulative effects of the entire 4-week drought treatment and reflect structural investment, whereas antioxidant and biochemical parameters capture the instantaneous state at the single end-of-treatment harvest. Therefore, the apparent trade-off between morphological and biochemical responses should be interpreted as temporal complementarity rather than a direct negative trade-off, because the two categories cannot be directly compared at a single time point. We would require repeated time-course sampling throughout the stress period to resolve the full dynamics between cumulative and instantaneous responses.

### Practical implications and limitations

These results carry practical implications for cultivar selection: ZD is a candidate for regions with variable or moderate water availability, whereas GE is better suited to irrigated systems. However, several limitations temper this conclusion. The confined PVC-tube system may not capture field root profiles and water dynamics, the single end-of-treatment harvest does not address regrowth after cutting, and forage yield, quality, and regrowth capacity were not measured. Cultivars were selected by climatic origin rather than prior screening. Field validation across multiple growing seasons is therefore required before these greenhouse-derived patterns are translated into agronomic recommendations. Future work should incorporate cell-type-specific transcriptomics (Ali et al., 2026) to resolve the signaling networks linking water-deficit perception to differential recruitment of the CAT/APX and GPX/GR pathway. In addition, only three cultivars were used, which limits the generality of our conclusions; validation with a broader germplasm panel is warranted. Root construction cost (e.g., carbon/nitrogen content or respiration-based construction cost) and root hydraulic properties were not measured, and antioxidant capacity was assayed on the whole root system without separating fine and coarse roots; these are valuable complements for future studies to link root morphological investment and water uptake to its metabolic cost under drought. Furthermore, our phenotypic assessments were performed on only four individual plants per cultivar-treatment combination. As alfalfa is an autotetraploid outcrossing species with substantial within-cultivar genetic heterogeneity, apparent cultivar-level differences may partly arise from sampling variation. Future studies should employ bulked samples comprising no fewer than 20 plants per cultivar to enhance the statistical robustness of inter-cultivar comparisons.

## Conclusion

This study demonstrates that alfalfa drought adaptation is cultivar-specific and arises from the concurrent regulation of root system architectural plasticity and antioxidant defense capacity. Under a controlled drought gradient, ZD exhibited a more balanced strategy, combining moderate root system expansion with balanced deployment of enzymatic (CAT, GPX) and non-enzymatic (AsA) antioxidants, resulting in lower oxidative damage and sustained biomass. AH showed strong root morphological plasticity but limited biochemical protection, whereas GE exhibited pronounced root responses with a narrower antioxidant repertoire and growth optimized under well-watered conditions. Water management and cultivar selection should account for these genotype-specific response patterns: ZD is recommended for variable or moderate water availability and GE for irrigated systems, pending field experiments. These findings provide a framework for integrating whole-root morphological and biochemical traits in alfalfa drought-tolerance screening and breeding.

## Data Availability

The original contributions presented in the study are included in the article/**Supplementary Material**. Further inquiries can be directed to the corresponding author.

## Supplementary material

Supplementary Table 1 Variable loadings on PC1 and PC2 and percentage contributions, sorted by PC1 contribution.

Supplementary Figure 1 The root system of three alfalfa cultivars under four soil moisture treatments.
