# Supplementary material for: Cultivar-specific coordination of root plasticity and root antioxidant defense underlies drought adaptation in alfalfa
Source: Front Plant Sci. 2026 Oct 8;17:1941938. doi: 10.3389/fpls.2026.1941938 (PMC13686840; doi:10.3389/fpls.2026.1941938)
Supplement: Supplementary file 1 [file SupplementaryFile2.docx]

Supplementary Figures

Severe drought	Moderate drought	Mild drought	Well-water

30%-35% WHC	45%-50%WHC	60%-65%WHC	75%-80%WHC

AH

ZD

GE

Figure. S1. The root system of three alfalfa cultivars under four soil moisture treatments.
