# Supplementary material for: Cultivar-specific coordination of root plasticity and root antioxidant defense underlies drought adaptation in alfalfa
Source: Front Plant Sci. 2026 Oct 8;17:1941938. doi: 10.3389/fpls.2026.1941938 (PMC13686840; doi:10.3389/fpls.2026.1941938)
Supplement: Supplementary file 2 [file SupplementaryFile3.docx]

Supplymentary Table

Table S1 Variable loadings on PC1 and PC2 and percentage contributions, sorted by PC1 contribution.

| Variable | PC1 loading | PC2 loading | PC1 contributions (%) | PC2 contributions (%) |
| --- | --- | --- | --- | --- |
| Root biomass | -0.306 | -0.007 | 9.37 | 0.01 |
| Root/shoot ratio | -0.301 | 0.130 | 9.03 | 1.69 |
| GPX | 0.300 | 0.159 | 9.02 | 2.54 |
| GR | 0.300 | -0.110 | 9.01 | 1.22 |
| O_2_^−^ | -0.295 | -0.176 | 8.68 | 3.09 |
| CAT | -0.278 | -0.306 | 7.74 | 9.36 |
| Total root length | -0.266 | 0.131 | 7.09 | 1.71 |
| Root surface area | -0.264 | 0.134 | 6.95 | 1.79 |
| Root volume | -0.264 | -0.179 | 6.95 | 3.22 |
| MDA | -0.262 | -0.056 | 6.85 | 0.31 |
| APX | -0.225 | -0.436 | 5.07 | 19.00 |
| Shoot biomass | 0.210 | -0.359 | 4.42 | 12.91 |
| H_2_O_2_ | -0.195 | 0.414 | 3.82 | 17.17 |
| AsA | 0.175 | -0.470 | 3.05 | 22.11 |
| Average root diameter | -0.172 | -0.197 | 2.95 | 3.86 |
